# Cardiovascular Risk, Obesity, and Sociodemographic Indicators in a Brazilian Population

**DOI:** 10.3389/fpubh.2021.725009

**Published:** 2021-11-30

**Authors:** Nayla Cristina do Vale Moreira, Ibrahimu Mdala, Akhtar Hussain, Bishwajit Bhowmik, Tasnima Siddiquee, Virgínia Oliveira Fernandes, Renan M. Montenegro, Haakon E. Meyer

**Affiliations:** ^1^Department of Community Medicine and Global Health, Institute of Health and Society, University of Oslo (UiO), Oslo, Norway; ^2^Faculty of Medicine, Federal University of Ceará (FAMED-UFC), Fortaleza, Brazil; ^3^Faculty of Health Sciences, NORD University, Bodø, Norway; ^4^Department of General Practice, Institute of Health and Society, University of Oslo (UiO), Oslo, Norway; ^5^Centre for Global Health Research, Diabetic Association of Bangladesh, Dhaka, Bangladesh; ^6^International Diabetes Federation, Brussels, Belgium; ^7^Norwegian Institute of Public Health, Oslo, Norway

**Keywords:** cardiovascular risk (CVD), Framingham risk score (FRS), obesity, sociodemographic indicators, anthropometric markers

## Abstract

**Background and Aims:** Cardiovascular diseases (CVDs) are the leading cause of death globally and in Brazil. Evidence suggests that the risk of CVDs differs by race/ethnicity. Scarce information exists about the association between CVD risk, obesity indicators and sociodemographic characteristics in the Brazilian population.

**Objectives:** We aimed to assess the CVD risk following the Framingham risk score in relation to the population's sociodemographic profile. Further, we examined the association between anthropometric markers and risk of CVDs.

**Methods:** A total of 701 subjects aged ≥20 years from North-eastern Brazil were recruited randomly to participate in a population-based, cross-sectional survey. Age-adjusted data for CVD risk, sociodemographic characteristics, and anthropometric indices were assessed, and their relationships examined.

**Results:** High CVD risk (Framingham risk score ≥10%) was observed in 18.9% of the population. Males (31.9 vs. 12.5%) and older subjects (age ≥45 years: 68.9% vs. age <45 years: 4.2%) had significantly higher risk of CVDs, whereas those employed in manual labor showed lower risk (7.6 vs. 21.7%). Central obesity measures like waist-to-hip ratio and waist-to-height ratio were more strongly associated with predicted CVD risk than body mass index.

**Conclusions:** Our population had a high risk of CVDs using the Framingham risk score. Cost-effective strategies for screening, prevention and treatment of CVDs may likely reduce disease burden and health expenditure in Brazil. Central obesity measures were strongly associated with predicted CVD risk and might be useful in the clinical assessment of patients. Follow-up studies are warranted to validate our findings.

## Introduction

Cardiovascular diseases (CVDs) have reached epidemic proportions worldwide, with a greater impact in low- and middle-income countries (LMICs), including Brazil (1). In 2016, approximately 17.9 million people died from CVDs globally, mostly due to heart attack and stroke. Over 75% of these deaths have taken place in LMICs (2). CVDs are the leading cause of death in Brazil and responsible for the highest healthcare expenditure for hospital admissions (3).

Most CVDs are caused by a complex interaction of several modifiable risk factors, including tobacco use, physical inactivity, unhealthy diet, overweight and obesity, harmful use of alcohol, hypertension, diabetes, and dyslipidaemia (1). Recently, Brazil has experienced a rapid demographic and economic transition, resulting in profound changes in nutritional and lifestyle patterns. Industrialization, urbanization, an aging population, and increased prevalence of unhealthy habits have become root causes of the rising CVD burden in Brazil (3). Amongst these risk factors, obesity is an increasing concern. According to 2016 estimates, around 22% of Brazilian adults aged ≥ 18 years and 9% of adolescents aged 10–19 years were obese (4).

Overweight and obesity have been regarded as one of the leading factors for mortality, accounting for around 23% of the ischaemic heart disease burden (5, 6). Several anthropometric measures of general and central obesity have been applied to assess adiposity-related risk, including body mass index (BMI), waist circumference (WC), waist-to-hip ratio (WHR), and waist-to-height ratio (WHtR) (5). However, previous studies have found conflicting results regarding the usefulness of these different anthropometric indices (7–9). Moreover, even though most of the global burden of CVDs is in developing countries, the existing evidence is derived mainly from high-income countries (10). Since adiposity is highly heterogeneous with age, gender, and ethnicity (11), it remains unclear which anthropometric parameters are better correlated with the risk of CVDs in different populations (12).

Evidence suggests that the risk of CVDs differs by race/ethnicity (13). In Brazil, although several studies were conducted for the prevalence of cardiovascular risk factors, most of them have limitations due to potential selection bias and the use of self-reported data in the absence of confirmatory laboratory examinations (14). Moreover, few studies have compared the independent associations between the different anthropometric indices and CVD risk based on the recommended cut-off values (15). Scarce information exists in Brazil about the risk of CVDs and sociodemographic and anthropometric characteristics of the population. Thus, in this cross-sectional, population-based study, we aimed to investigate CVD risk following the Framingham risk score and how it is related to socioeconomic and demographic characteristics. We also studied the association between some anthropometric markers, i.e., WC, BMI, WHR and WHtR, and the predicted risk of CVDs in both genders.

## Materials and Methods

### Study Population

This cross-sectional study was carried out between August 2012 and January 2013 in the city of Pindoretama, located in the state of Ceara (CE), North-eastern Brazil. The recruitment and examination procedures have been discussed previously (16). According to the latest demographic census conducted in 2010, the total population of Pindoretama was approximately 18,683 inhabitants (17). The health registry list with the citizens' names in alphabetic order was applied to select the potential study subjects. Random numbers were generated with the statistical software R (18) and identified with the names in the list thereafter. The selected subjects were invited to participate in the survey by local Community Health Workers (CHW). Around 1,000 subjects were randomly selected based on the list. Of these, one hundred and sixty-three were not found by the CHW and, therefore, could not receive the invitation. Thirty-one subjects did not meet the inclusion criteria. Thus, eight hundred and six randomly selected subjects were invited, of whom 714 agreed to participate (a response rate of 88.6%).

Subjects of both genders, aged ≥ 20 years who were able to communicate and willing to participate in the study were considered eligible. Those with an acute or chronic severe cardiac, renal, or hepatic illness, as well as physically or mentally disabled subjects unable to follow simple questions and examinations were excluded, as were pregnant women. Since we aimed to assess the CVD risk, those with a previous history of myocardial infarction and/or stroke were considered as having had the condition, and therefore were excluded from the analyses (13 subjects). Seven hundred and one subjects remained. At the time of recruitment, the subjects were requested to visit a nearby health center after an overnight fast of 8–10 h. Pre-tested questionnaires were conducted by trained interviewers to collect sociodemographic and clinical information. Anthropometric measurements, blood pressure, and body fat percentage (BF%) were also registered.

### Sample Size Calculation

The required sample size was calculated by the formula: *n* = 4 (*z*_*crit*_)^2^ *p* (1–*p*)/*D*^2^ (19). The total sample size was represented by “*n*”, “*z*_*crit*_” = 1.96 (Standard Normal Deviate for a Significance Criterion = 0.05 and a Confidence Interval = 0.95), “*p*” = 0.051 prevalence estimate from a previous study of high/intermediate risk of CVD according to the Framingham risk score (20), and “*D*” = 0.0454 (total width of the expected confidence interval). Two-tailed statistical analyses were used. Thus, *n* = 4 × (1.96)^2^ × 0.051 × 0.949/(0.0454)^2^; *n* = 360.83.

### Ethics

The study was conducted according to the ethical principles outlined in the Helsinki Declaration (21). The research protocol was approved by the local Ethical Committee in Brazil (Protocol Number: 045.06.12) and the Regional Committee for Medical and Health Research Ethics (REK) in Norway (Reference: 2012/779/REK sør-øst D). Written or verbal consent was sought from each subject prior to any investigation. The subjects were informed of their right to withdraw from the study at any point or withhold their data from the analysis. Those who were diagnosed with any clinical condition were referred to the nearest health center for treatment and follow-up.

### Measurements

Weight, height, WC, and hip circumference (HC) were taken with subjects standing without shoes and wearing light clothing. Body weight (kilograms) was registered to the nearest 0.1 kg using a portable digital scale, calibrated before use and checked every day with a known weight. Height (centimeters) was measured by applying a well-mounted stadiometer, with each subject standing upright with their head in the Frankfurt plane. BMI was calculated as the weight in kilograms divided by the square of the height in meters (kg/m^2^). BF% was determined by a portable bipolar body fat analyser (Omron®, Model HBF-306, Omron Healthcare, Inc., Illinois, United States). WC was measured with a non-stretchable tape, positioned horizontally in the middle area between the lower border of the ribs and iliac crest, under the mid-axillary line. HC was assessed with the same tape positioned to the maximum circumference around the buttocks, with the subjects standing straight. WC and HC were recorded to the nearest 0.1 cm. WHR was calculated as the WC divided by the HC, while the WHtR as the WC divided by the height.

Blood pressure (mmHg) was estimated twice at a 10-min interval using a validated automatic sphygmomanometer (Omron® BP785 IntelliSense® Automatic Blood Pressure Monitor with ComFitTM Cuff, Omron Healthcare, Inc., Illinois, United States), with appropriate cuffs, in a sitting position after a resting time of at least 15 min. The mean of the two readings was used for the analysis.

### Blood Sampling and Laboratory Assays

On arrival at the data collection center, a 10-mL fasting venous blood sample was taken to determine the concentrations of plasma glucose, insulin, and lipids. Two hours after a 75g oral glucose load, another 3 ml of venous blood was drawn for the oral glucose tolerance test (OGTT). Blood samples were stored immediately over ice and centrifuged after 1 h. Plasma was frozen and transported to the laboratory, where the samples were stored at −20 °C until the analyses were conducted. Quality control of the laboratory was assessed internally and externally.

The glucose oxidase method was applied to estimate fasting and 2-h plasma glucose levels, whereas fasting insulin was determined by chemiluminescence. Total cholesterol (TC) was assessed by the cholesterol oxidase - phenol + aminophenazone (CHOD-PAP) method, while a homogenous enzymatic colorimetric method was used to determine high-density lipoprotein cholesterol (HDL-C) levels. Triglycerides (TG) were assessed by the glycerol-3-phosphate oxidase - phenol + aminophenazone (GPO-PAP) method. The Friedewald Formula (22) was applied to calculate the low-density lipoprotein cholesterol (LDL-C) levels.

### Definitions of Variables

The cut-off points for WHR recommended by the World Health Organization (WHO) were applied, i.e., for males, a WHR ≥ 0.90 was classified as “high” (substantially increased risk of metabolic complications), whereas, for females, a WHR ≥ 0.85 was considered “high”. Overweight/obese was defined by a BMI of ≥ 25 kg/m^2^. A high WC was described as > 102 cm for males, and > 88 cm for females (23). A cut-off of ≥ 0.50 was applied to define a high WHtR (24).

Following the definition of the Brazilian Institute of Geography and Statistics (IBGE) classification, ethnicity was assessed according to the subjects' self-perception of their skin color. The different ethnic groups were categorized into “white” and “non-white” (17). Physical activity information was ascertained by the International Physical Activity Questionnaire (IPAQ) short form (25). The IPAQ's total score was computed by summing up the duration and frequency of walking, moderate- and vigorous-intensity activities. Following the guidelines for data processing and analysis, the levels of physical activity were classified into “low”, “moderate” and “high” (26). We further categorized them into “low” and “moderate” plus “high” level. Current smoking included those who self-reported as being smokers or had stopped smoking for less than 1 year. Alcohol consumption was ascertained by self-report as yes/no. The occupation of the subjects was categorized into manual and non-manual labor. Manual labor was used to describe jobs in agriculture and construction, whereas non-manual labor described all other occupations.

The 1999 WHO criteria (27) were applied in diagnosing diabetes mellitus. Diabetes cases were defined as those who had a previous diagnosis of type 2 diabetes, or those with fasting (venous) plasma glucose value ≥ 7.0 mmol/l (≥ 126 mg/dl), or the 2-h plasma glucose value after a 75 g oral glucose load ≥ 11.1 mmol/l (≥ 200 mg/dl), or both. Dyslipidaemia was defined as TG ≥ 1.7 mmol/l and HDL <0.9 mmol/l for males; and <1.0 mmol/l for females (27). Insulin resistance was estimated by the homeostasis model assessment of insulin resistance (HOMA-IR = [insulin (mU/l) × glucose (mmol/l)] / 22.5) (28). Hypertension was defined as systolic blood pressure (SBP) ≥ 140 mmHg and/or diastolic blood pressure (DBP) ≥ 90 mmHg and/or being on blood-pressure-lowering medication (29).

### Estimating the Framingham Risk Score

**Figure 1:**
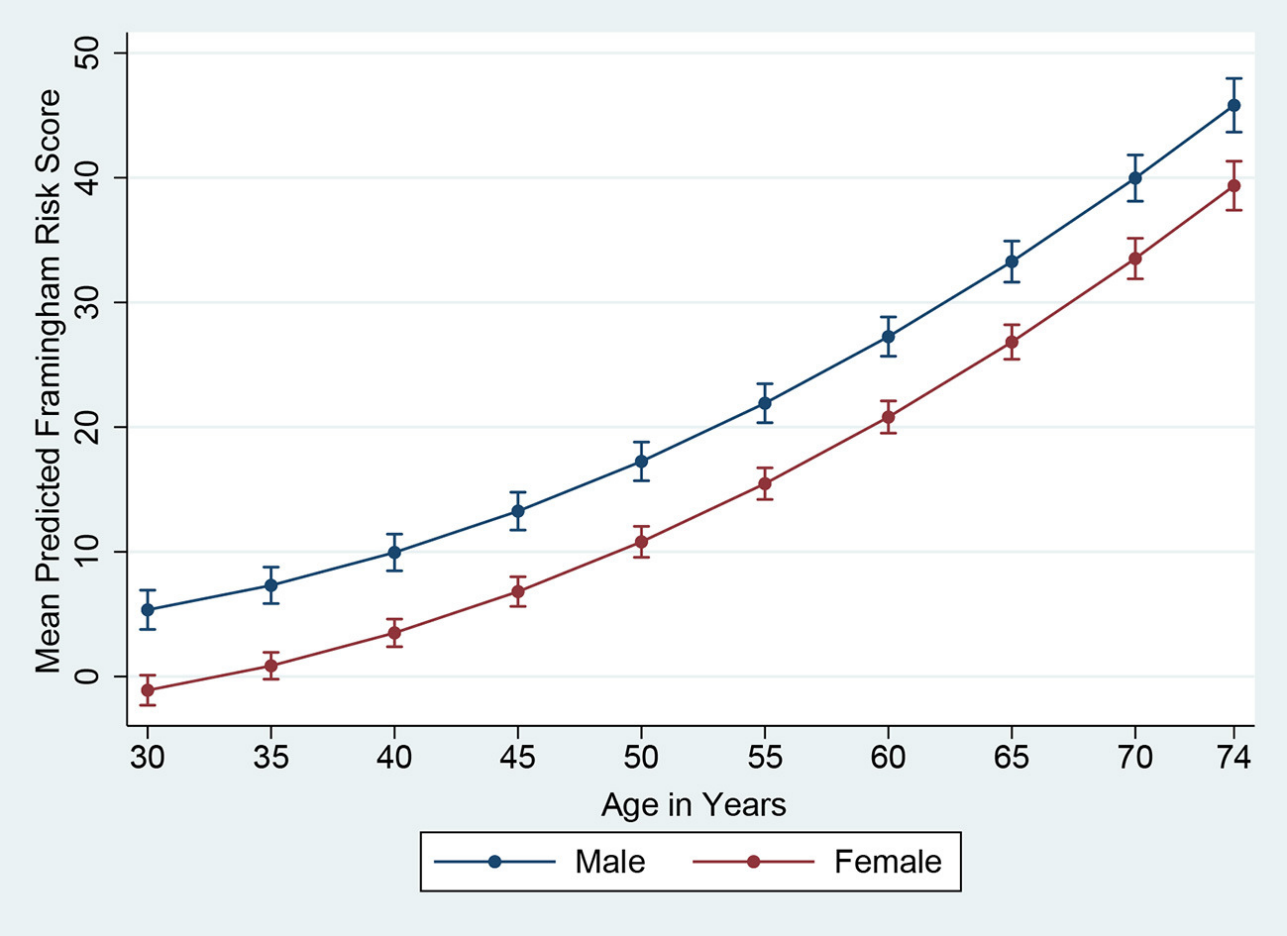
Framingham Risk Score by age and gender (vertical lines are means with 95% CIs).

**Figure 2:**
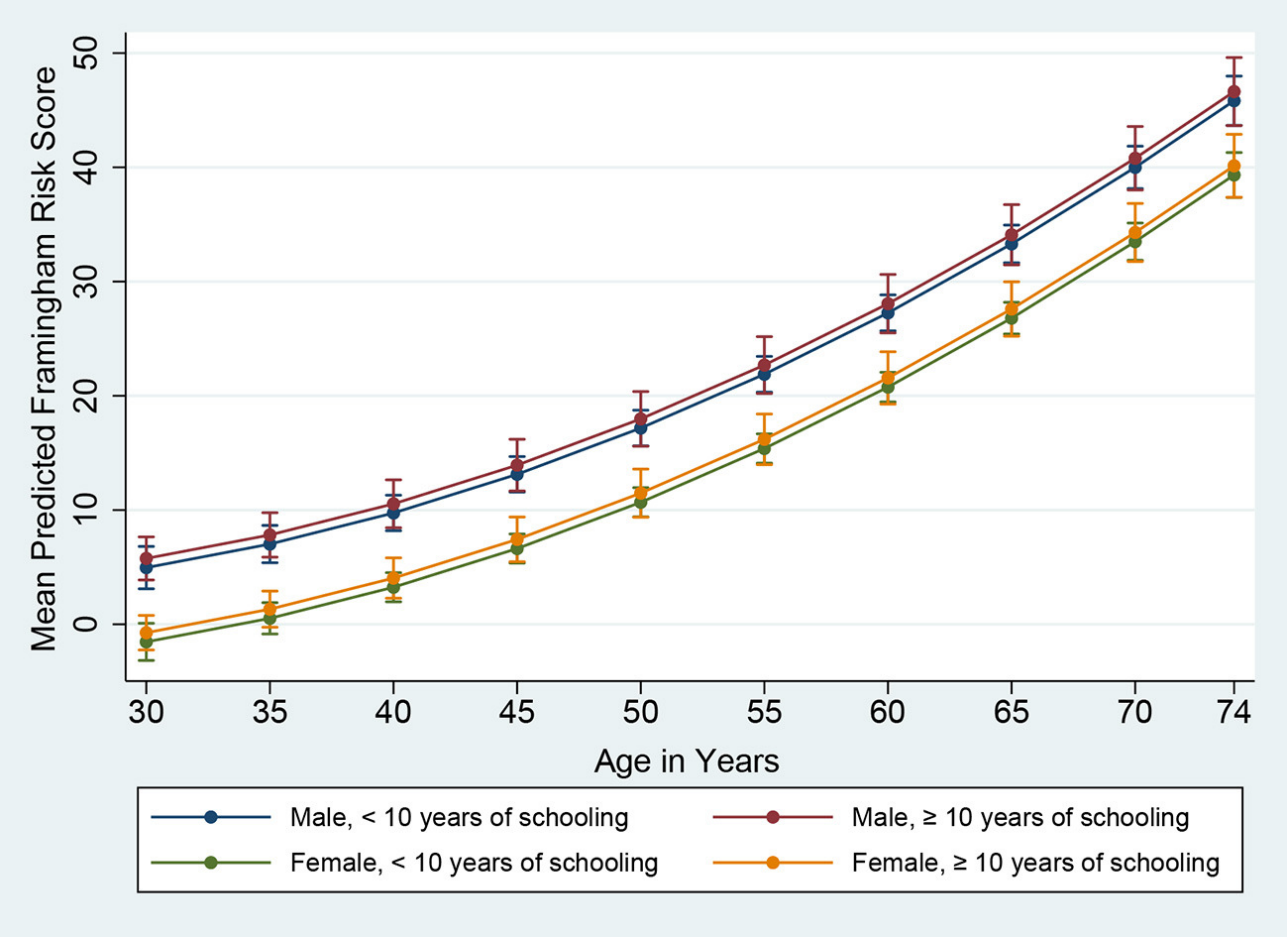
Framingham Risk Score by age, gender, and education (vertical lines are means with 95% CIs).

The Framingham 10-year risk score model, as published by D'Agostino et al. (30) in 2008, was applied to estimate the predicted 10-year risk for an incident cardiovascular event. The model predictors included age, gender, SBP, use of antihypertensive medication, TC, HDL-C, smoking and diabetes status (30). Subjects with a Framingham predicted risk of 10% or above during the next 10 years were defined as having high CVD risk. Although data were collected from 701 subjects in total, the Framingham risk score was estimated for 693 subjects owing to missing values (229 males and 464 females). In Figures 1, 2, based on D'Agostino et al. (30) and Chang et al. (31), we presented the mean Framingham risk score estimates for the age range 30–74 years.

### Statistical Analysis

Continuous data were expressed as means and 95% confidence intervals (CIs), while percentages and 95% CIs were given for categorical variables. Generalized linear regression models (GLM) were fitted to the data after adjusting for age. To compare differences between adjusted means, we fitted GLMs with linear link function, while GLMs with the logit link function were applied to compare differences between proportions. The prevalence of those with a predicted 10-year CVD risk of ≥ 10% was calculated as predictive margins, based on the estimation of the adjusted logistic regression models. To control confounding by age in the predicted means and prevalence, we fixed age at 45 years, which was the closest to the mean age in the sample. A two-sample test of proportions was applied to compare the prevalence of high CVD among the different sociodemographic groups. Anthropometric measurements were converted to z-scores [original value subtracted by the mean and divided by the standard deviation (SD)] to represent the number of SDs above and below the mean for each subject. Multiple linear regression was carried out to investigate the relationship between the standardized anthropometric markers and CVD risk. Further, we calculated crude and adjusted prevalence ratios (PRs) of the anthropometric indicators for detecting high CVD risk using Poisson regression with robust variance, as the prevalence of high CVD risk was above 10%. We tested for two-way interaction between age and the different anthropometric markers. The Akaike Information Criteria (AIC) was used to compare nested models. Data were analyzed using Stata 15^th^ edition (32) and SPSS 26^th^ version (33) statistical software. The results were considered statistically significant with *p* < 0.05, and all tests were two-sided.

## Results

A total of 701 subjects (234 males and 467 females, mean age 44.8 ± 16.0 SD) were included in the analysis. Significant differences were found in sociodemographic, lifestyle, anthropometric and cardiometabolic characteristics between the genders (Table 1). Males had a significantly higher proportion of tobacco smoking and alcohol consumption. Females were more physically inactive and showed a higher percentage of overweight/obesity. Age, dyslipidaemia, hypertension, and diabetes status did not differ between the genders. Anthropometric parameters including mean HC, WHtR, BMI and BF% were higher in females, while males had a higher WHR.

**Table 1:** Baseline characteristics of the study subjects.

**Characteristics**	**All (*n* = 701)**	**Males(*n* = 234)**	**Females (*n* = 467)**	*p*–value*
Age (years)	44.8 (43.6–46.0)	45.6 (43.6–47.7)	44.4 (42.9–45.8)	0.319
Ethnicity (%)				
White	16.6% (13.8–19.3)	10.7% (6.7–14.6)	19.5% (15.9–23.1)	0.003
Non–white	83.4% (80.7–86.2)	89.3% (85.4–93.3)	80.5% (76.9–84.1)	
Education (%)				
<10 years	79.9 (76.0–83.8)	84.7 (79.7–89.7)	77.5 (72.8–82.2)	0.024
≥10 years	20.1 (16.2–24.0)	15.3 (10.3–20.3)	22.5 (17.8–27.2)	
Monthly Income (%)				
<2MW	90.2 (88.0–92.4)	80.6 (75.5–85.7)	95.0 (93.0–96.9)	<0.001
≥2MW	9.8 (7.6–12.0)	19.4 (14.3–24.5)	5.0 (3.1–7.0)	
Manual Labor (%) **	9.5 (7.5–11.4)	27.5 (21.7–33.3)	0.4 (−0.2 – 1.0)	<0.001
Currently Married (%)	66.7 (63.3–70.2)	74.3 (68.8–79.9)	62.9 (58.5–67.3)	0.003
Smoking (yes) (%) ***	38.8 (35.0–42.7)	48.6 (41.7–55.5)	33.9 (29.3–38.5)	<0.001
Alcohol Consumption (yes)	35.1 (31.5–38.6)	54.2 (47.5–61.0)	25.4 (21.3–29.6)	<0.001
Physical Activity (%)				
Low	66.8 (63.4–70.3)	55.4 (49.0–61.8)	72.6 (68.5–76.6)	<0.001
Moderate/High	33.2 (29.7–36.6)	44.6 (38.2–51.0)	27.4 (23.4–31.5)	
WC (cm)	90.1 (89.2–91.0)	89.6 (88.0–91.2)	90.4 (89.3–91.5)	0.415
HC (cm)	98.6 (97.9–99.4)	95.7 (94.4–97.0)	100.1 (99.2–101.0)	<0.001
WHR, mean	0.92 (0.91–0.92)	0.94 (0.93–0.95)	0.90 (0.89–0.91)	<0.001
WHtR, mean	0.57 (0.56–0.58)	0.54 (0.53–0.55)	0.59 (0.58–0.60)	<0.001
BMI (kg/m^2^)	26.9 (26.5–27.3)	25.9 (25.3–26.6)	27.4 (26.9–27.8)	<0.001
Overweight/Obese (%)	61.9 (58.4–65.5)	53.4 (47.0–59.8)	66.2 (61.9–70.5)	0.001
BF%, mean	32.8 (32.3–33.4)	24.8 (23.9–25.7)	36.9 (36.2–37.5)	<0.001
SBP (mmHg)	127.6 (126.2–129.0)	132.7 (130.3–135.1)	125.1 (123.4–126.8)	<0.001
DBP (mmHg)	76.8 (75.6–78.1)	77.7 (75.5–79.9)	76.4 (74.8–77.9)	0.326
Hypertension (%)	29.8 (25.8–33.8)	29.3 (22.6–36.1)	30.1 (25.2–34.9)	0.863
Diabetes (%)	14.3 (11.6–17.0)	11.7 (7.6–15.8)	15.6 (12.2–19.0)	0.159
HOMA–IR	1.6 (1.5–1.7)	1.3 (1.1–1.5)	1.8 (1.7–1.9)	<0.001
Total Cholesterol (mmol/l)	4.72 (4.65–4.79)	4.62 (4.50–4.75)	4.76 (4.68–4.85)	0.069
HDL (mmol/l)	1.22 (1.21–1.23)	1.23 (1.21–1.24)	1.22 (1.21–1.23)	0.475
LDL (mmol/l)	2.86 (2.80–2.93)	2.74 (2.63–2.86)	2.92 (2.84–3.00)	0.014
Triglycerides (mmol/l)	1.54 (1.41–1.67)	1.75 (1.52–1.97)	1.44 (1.28–1.60)	0.031
Dyslipidaemia (%)	24.8 (21.6–28.0)	24.2 (18.7–29.8)	25.0 (21.1–29.0)	0.817

Data are mean (95% confidence intervals) or percentage (95% confidence intervals). Model was evaluated at age 45 years; *p-value for the difference between males and females. **Manual Labor = refers to jobs in agriculture and construction; ***Included those who self-reported as being smokers or had stopped smoking for less than 1 year. BF%, Body Fat Percentage; BMI, Body Mass Index; DBP, Diastolic Blood Pressure; HC, Hip Circumference; HDL-C, High-Density Lipoprotein Cholesterol; HOMA-IR, Homeostasis Model Assessment of Insulin Resistance; LDL-C, Low-Density Lipoprotein Cholesterol; MW, Minimum Wage in 2012; SBP, Systolic Blood Pressure; WC, Waist Circumference; WHtR, Waist-to-Height Ratio; WHR, Waist-to-Hip Ratio.

The mean predicted Framingham risk score increased substantially with age and was higher among males (Figure 1). In addition, the mean predicted risk was not statistically significant between different levels of education in both genders (Figure 2).

As shown in Table 2, the estimated proportion with a predicted 10-year CVD risk of ≥ 10% was significantly higher among males (31.9 vs. 12.5%; *p*-value: < 0.001), and those with more than 45 years of age (68.9 vs. 4.2%; *p*-value: < 0.001). Furthermore, it was significantly lower among those with an occupation requiring manual labor (7.6 vs. 21.7%; *p*-value: 0.008), defined as jobs in agriculture and construction.

**Table 2:** Predicted proportions of subjects with 10-year CVD risk of ≥10% using the Framingham Risk Score by sociodemographic characteristics.

**Characteristics**	**n**	**Predicted 10–year risk ≥10% *% (95% CIs)***	***p*–value**
Overall	693*	18.9 (14.3–23.6)	
**Gender**			
Male	229	31.9 (21.8–42.0)	<0.001
Female	464	12.5 (8.0–17.0)	
**Age groups**			
<45 years	388	4.2 (2.2–6.2)	<0.001
≥45 years	305	68.9 (63.8–74.0)	
**Ethnicity**			
White	116	20.8 (9.4–32.2)	0.58
Non-white	577	18.6 (13.7–23.5)	
**Education**			
<10 years	489	19.2 (14.1–24.4)	0.60
≥10 years	204	17.5 (7.4–27.7)	
**Monthly income**			
<2MW	623	17.7 (12.8–22.7)	0.11
≥2MW	68	25.6 (11.4–39.9)	
Occupation**			
Non-manual labor	629	21.7 (16.4–27.1)	0.008
Manual labor	64	7.6 (1.3–13.9)	

Data are percentage (95% confidence intervals), adjusted for age (at age fixed to 45 years) and gender. *The study collected data from 701 subjects in total, but due to some missing values, the Framingham Risk Score was calculated for 693 subjects (229 males and 464 females). **Manual Labor: jobs in agriculture and construction. Non-manual Labor: other occupations. CIs: Confidence Intervals. CVD: Cardiovascular Disease. MW: Minimum Wage in 2012.

Multiple linear regression was carried out to assess the age-adjusted associations between 1 SD increment in each anthropometric marker and the predict risk of CVDs, entered as a continuous variable (Table 3). In males, only WHtR was a significant predictor of CVD risk followed by a borderline-significant association for WC, while in females WHR and WHtR were statistically significant. WHtR showed the highest slope coefficient in males, whereas WHR had the highest slope in females.

**Table 3:** Association between 1 SD increase in anthropometric markers and CVD risk, using the Framingham Risk Score, age adjusted.

**Characteristics**	**Slope coefficient (β) (95% CIs)**	*p*–value^*^	**R square**
**Males**			
WC (per 1 SD)	1.67 (−0.01–3.35)	0.05	0.6754
BMI (per 1 SD)	1.56 (−0.06–3.19)	0.06	0.6750
WHR (per 1 SD)	1.30 (−0.48–3.09)	0.15	0.6728
WHtR (per 1 SD)	1.82 (0.09–3.56)	0.04	0.6760
**Females**			
WC (per 1 SD)	0.78 (−0.12–1.69)	0.09	0.5811
BMI (per 1 SD)	0.60 (−0.27–1.48)	0.18	0.5801
WHR (per 1 SD)	1.13 (0.14–2.11)	0.03	0.5830
WHtR (per 1 SD)	0.95 (0.01–1.89)	0.04	0.5820

* *p-value for each predictor in the regression model, controlling for age. BMI, Body Mass Index; Cis, Confidence Intervals; CVD, Cardiovascular Disease; SD, Standard Deviation; WC, Waist Circumference; WHR, Waist-to-Hip Ratio; WHtR, Waist-to-Height Ratio*.

Table 4 presents the PRs of the different anthropometric measures for identifying a high CVD risk. Univariable and multivariable Poisson regression analyses with robust variance were applied. Adjusted PRs were obtained after controlling for age, level of physical activity, family history of cardiac disease and stroke. An interaction term between age and the corresponding anthropometric parameter was included in some adjusted models, according to their statistical significance and the Akaike Information Criteria (AIC). In females, significant positive associations were found between all anthropometric variables and high CVD risk in the adjusted models, except for WC. In males, all anthropometric markers were significant. WHtR had the highest adjusted PR for males (9.9, 95% CI: 2.8–34.8, *p*-value < 0.001) and females (43.4, 95% CI: 2.6–716.8, *p*-value 0.002). This large PR and wide CI in the adjusted model for females and WHtR was due to the few observations of those with high CVD risk and WHtR <0.50 (6 subjects).

**Table 4:** Crude and adjusted prevalence ratios (PRs) of anthropometric indices for identifying high CVD risk (≥10% using the Framingham Risk Score).

**Characteristics**	**Crude PR^**a**^ (95% CIs)**	***p–*value**	**Adjusted PR^**b**^ (95% CIs)**	***p*–value**
**Males**				
WC (>102 cm)^c^	1.9 (1.4–2.5)	<0.001	7.5 (2.1–27.0)	0.002
BMI (≥25 kg/m^2^)^c^	1.2 (0.8–1.6)	0.366	4.9 (1.6–14.9)	0.005
WHR (≥0.90)^c^	2.7 (1.7–4.2)	<0.001	8.7 (2.4–31.5)	0.001
WHtR (≥0.50)^c^	2.3 (1.4–3.7)	0.001	9.9 (2.8–34.8)	<0.001
**Females**				
WC (> 88 cm)	1.7 (1.2–2.3)	0.001	1.3 (1.0–1.7)	0.087
BMI (≥25 kg/m^2^)	1.1 (0.8–1.5)	0.565	1.4 (1.1–1.9)	0.008
WHR (≥0.85)^c^	4.1 (2.4–7.3)	<0.001	11.0 (2.8–43.6)	0.001
WHtR (≥0.50)^c^	3.5 (1.6–7.6)	0.002	43.4 (2.6–716.8)	0.008

a *Crude prevalence ratio after univariable Poisson regression analysis*.

b *Adjusted prevalence ratios for age, level of physical activity, family history of cardiac disease and stroke*.

c *An interaction term between each anthropometric marker and age was included in the adjusted models. The Akaike Information Criteria (AIC) was used to compare nested models. BMI, Body Mass Index; Cis, Confidence Intervals; CVD, Cardiovascular Disease; WC, Waist Circumference; WHR, Waist-to-Hip Ratio; WHtR, Waist-to-Height Ratio*.

## Discussion

To the best of our knowledge, this is one of the few population-based studies from Brazil to investigate the CVD risk by sociodemographic characteristics, as well as the association between different obesity markers and the risk of a cardiovascular event. Males and older people presented higher risk of CVDs in our population, whereas those employed in the manual labor had significantly lower risk. Central obesity measures were more strongly associated with CVD risk than BMI.

We found a high prevalence of increased CVD risk, i.e., Framingham risk score ≥ 10%, in this population. Our estimates were higher than those reported in Peru (34), Argentina (35) and Southern Brazil (20, 36), similar to India (37), but lower than Honduras (38) and China (39). These differences might be explained by genetic, racial, sociodemographic, and cultural diversity, as well as the use of other versions of the Framingham risk score, with a varied set of predictors. In our sample, the prevalence of diabetes, smoking, hypertension, and dyslipidaemia was higher than reported in some other Brazilian surveys (3). The recent rapid industrialization and urbanization of Pindoretama (the rural population decreased from 66% to 39% between 1991 and 2010) (40), resulting in lifestyle and dietary changes, might explain the frequent occurrence of these cardiovascular risk factors and subsequent high Framingham risk score in the studied population.

Consistent with previous research (35, 37, 41, 42), males had a higher Framingham risk score than females. This might be due to the significantly higher SBP, and tobacco use among males. As expected, the Framingham risk score increased significantly with age, which is also in line with other studies (36, 41). The subjects employed in in agriculture or construction showed lower CVD risk, possibly reflecting the protective effect of physical activity (1). After controlling for age and gender, among those employed in manual labor, about 60.4% presented a moderate to high level of physical activity, whereas only 30.5% of those in other employment categories were similarly active (data not shown). On the other hand, the CVD risk did not differ significantly among the ethnic groups. This might be explained by the mixed genetic composition of the Brazilian population, essentially formed by an admixture of native Brazilians, Europeans, and Africans (43). It is likely that the extensive miscegenation of the overall Brazilian population may have reduced the differences among the ethnic groups. Although other studies have found an inverse relationship between CVD risk and education, our data did not find significant results. The relationship between education and risk of CVDs has shown great variability across populations, depending particularly on the level of health transition and socio-economic development of the country (31, 44). In 2010, the illiteracy rate in Pindoretama among those aged 15 and older was approximately 22%, whereas in Brazil it was 10% (40). In our data, only 4% of the subjects had a university degree or higher (data not shown). Therefore, it is likely that the lack of significant association between education and CVD risk might be due to the overall low level of education in our sample.

We found that the adjusted PR for WHR and WHtR were the highest among the anthropometric indices in relation to increased CVD risk. Further, the association between the WHR and Framingham risk score entered as a continuous variable was higher than that of WC, BMI and WHtR in females, whereas the slope coefficient of WHtR was the highest in males followed by WC. These results may indicate that these central obesity measures were more predictive of CVD risk than the general obesity measure like BMI. Therefore, in line with several others (5, 10), our findings suggest that BMI alone is insufficient to account for the association between CVD risk and obesity in this population. Over recent years, accumulating evidence has shown that abdominal obesity is more strongly associated with metabolic and cardiovascular problems than total adiposity (10, 45). Even within normal ranges of BMI, high visceral fat deposition remains an independent cardiovascular risk factor (45). Although BMI is strongly correlated with gold standard body fat measures, it cannot distinguish between lean and fat mass and does not delineate body fat distribution patterns (46). Whilst BMI would not account for an increase in muscle or fat-free mass, this would be reflected in the central obesity measures (5). Accumulation of visceral fat is related to insulin resistance, increased systemic inflammation, accelerated progression of atherosclerosis and endothelial dysfunction, which contribute to CVD risk (5, 45). This might explain the stronger association between abdominal obesity measures and CVD risk reported in our study. Generally, measures of obesity are not included in the prediction of CVD risk (5). Considering our findings, it might be beneficial to incorporate central obesity indicators such as WHR and WHtR into the clinical assessment of CVD risk.

Some studies have identified WC as the most highly correlated marker with CVD risk factors compared with other central obesity measures and BMI in females (47). Nevertheless, another cross-sectional study from Brazil including 270 women also reported that WHR showed a greater performance than WC in discriminating high coronary risk (15). Although WHR is more difficult to measure than WC, it has been considered a more specific surrogate for fat distribution, presents high precision and no bias over several ethnic groups (5). Our study showed a strong association between WHtR and CVD risk. Contrary to our results, a systematic review and meta-analysis reported that WHtR had the weakest association with CVD risk factors, compared with BMI and other measures of central obesity (47). However, other studies showed an opposite scenario in which WHtR was the most highly correlated obesity marker with CVD risk (5). Compared to WC, studies in different populations have described WHtR as a more sensitive indicator, possibly due to the adjustment to different statures and negative correlation of height to some metabolic risk factors (48).

This study contributes to the limited body of evidence from Brazil on CVD risk and sociodemographic characteristics, as well as on the association between different obesity measures and the risk of CVDs. The subjects were randomly selected, and the participation rate was high. The survey was performed by trained personnel and pre-tested questionnaires were applied. To minimize the risk of misclassification errors due to poor recall, anthropometric parameters were carefully assessed, and no self-reported measures were used. Blood samples were collected, handled, and transported according to standard protocols. Quality control of the laboratory was assessed internally and externally.

Our study had some limitations. It was based on a cross-sectional design and the 10-year CVD risk was calculated instead of using prospective CVD events. Nevertheless, the study generated valuable epidemiological data from Brazil regarding the association between CVD risk, obesity indicators and sociodemographic characteristics. Considering that Brazil is a large country with marked socioeconomic, ethnic, and regional disparities, our findings may not be representative for the whole nation. Caution should be taken when generalizing the results. However, since Brazilians have a mixed background, our sample might be a fair representation of the country's population. Furthermore, we had an overrepresentation of females (females 467 vs. males 234). As previously mentioned, out of 1,000 names randomly selected from the healthy registry list, around 163 subjects were not found by the CHW and therefore could not be invited to participate in the study. Out of these 163, approximately 78% were males. Additionally, among 92 subjects who refused to participate, around 63% were males. Population-based studies conducted during the day may constitute a hindrance to male participation. Males are often involved in income-generating work and therefore may not have been able to participate in the survey. The overrepresentation of females was dealt with by adjusting the analyses for gender or stratifying by gender. The Framingham risk score was not recalibrated for our population, which might have introduced some uncertainty in the CVD risk estimation. However, this was beyond the scope of the study and our available resources. Furthermore, the Framingham risk score has been widely applied and validated in ethnically diverse cohorts including whites, blacks, Native Americans, and Hispanics (49).

A high risk of CVDs according to the Framingham risk score was found in this population, especially among males and older people. In addition, manual labor seems to provide a protective effect on CVD risk. A timely targeted investment in screening, prevention, and necessary treatment of CVDs could reduce the burden on many and reduce the pressure on the health budget. Central obesity measures are more strongly associated with CVD risk than general obesity indicators. Our data suggest that WHR and WHtR are the best anthropometric markers to identify high CVD risk. Since an increase in muscle mass might not lead to changes in BMI (5), central obesity markers might be more useful to evaluate the effect of lifestyle changes in relation to CVD risk. Therefore, measuring WHR or WHtR might be beneficial in the clinical assessment of CVD risk. Prospective studies are still needed to further elucidate future risk of CVDs and their relationship with obesity in Brazil.

## Data Availability Statement

The raw data supporting the conclusions of this article will be made available by the authors, without undue reservation.

## Ethics Statement

The studies involving human participants were reviewed and approved by the Local Ethical Committee in Brazil (Protocol Number: 045.06.12) and the Regional Committee for Medical and Health Research Ethics (REK) in Norway (Reference: 2012/779/REK sør-øst D). The patients/participants provided their written informed consent to participate in this study. In case of illiteracy, verbal consent was assured by a local witness, who signed the informed consent, to secure the free participation of the subjects.

## Author Contributions

NM contributed to the conceptualisation of research goals and design, drafting of the article, as well as acquisition, analysis, and interpretation of the data. AH contributed to the study concept and design, data analyses, writing the initial draft and revising it critically, study oversight, and leadership. BB participated in the analysis and interpretation of data, writing the article, and revising it critically. IM took part in conceptualizing the study aims and design, writing the initial draft and revising it critically, as well as organizing the database, and conducting the statistical analysis. TS contributed to the design of the study, data curation and analyses, and drafting of the article. VF contributed to conceptualizing the work and designing the methodology, data collection and analyses, writing the initial draft, and revising it critically. RM contributed to the study concept and design, data analyses, study management and coordination, as well as writing the initial draft, and revising it critically. HM contributed to the design of the methodology, data analyses, study oversight and leadership, writing the initial draft, and performing critical review. All authors read and approved the content of the final manuscript. Furthermore, they agreed to be accountable for all aspects of the work in ensuring that questions related to the accuracy or integrity of any part of the work are appropriately investigated and resolved.

## Funding

This study was funded by the Ivar Helles Foundation, and the Health Department of the city of Pindoretama-CE.

## Conflict of Interest

The authors declare that the research was conducted in the absence of any commercial or financial relationships that could be construed as a potential conflict of interest.

## Publisher's Note

All claims expressed in this article are solely those of the authors and do not necessarily represent those of their affiliated organizations, or those of the publisher, the editors and the reviewers. Any product that may be evaluated in this article, or claim that may be made by its manufacturer, is not guaranteed or endorsed by the publisher.
